# Photophysics of sunscreen molecules in the gas phase: a stepwise approach towards understanding and developing next-generation sunscreens

**DOI:** 10.1098/rspa.2016.0677

**Published:** 2016-11

**Authors:** Natércia D. N. Rodrigues, Michael Staniforth, Vasilios G. Stavros

**Affiliations:** Department of Chemistry, University of Warwick, Coventry CV4 7AL, UK

**Keywords:** frequency- and time-resolved spectroscopy, ultrafast dynamics, photophysics, sunscreens, bottom-up approaches, increasing molecular complexity

## Abstract

The relationship between exposure to ultraviolet (UV) radiation and skin cancer urges the need for extra photoprotection, which is presently provided by widespread commercially available sunscreen lotions. Apart from having a large absorption cross section in the UVA and UVB regions of the electromagnetic spectrum, the chemical absorbers in these photoprotective products should also be able to dissipate the excess energy in a safe way, i.e. without releasing photoproducts or inducing any further, harmful, photochemistry. While sunscreens are tested for both their photoprotective capability and dermatological compatibility, phenomena occurring at the molecular level upon absorption of UV radiation are largely overlooked. To date, there is only a limited amount of information regarding the photochemistry and photophysics of these sunscreen molecules. However, a thorough understanding of the intrinsic mechanisms by which popular sunscreen molecular constituents dissipate excess energy has the potential to aid in the design of more efficient, safer sunscreens. In this review, we explore the potential of using gas-phase frequency- and time-resolved spectroscopies in an effort to better understand the photoinduced excited-state dynamics, or *photodynamics*, of sunscreen molecules. Complementary computational studies are also briefly discussed. Finally, the future outlook of expanding these gas-phase studies into the solution phase is considered.

## 1. Introduction

Despite efforts to raise awareness towards both the correct use of sunscreens and the risks of excessive sun exposure, skin cancer cases have risen in recent years [1–3]. According to the World Health Organization, 2–3 million non-melanoma and 132 000 melanoma skin cancers occur per year worldwide (as of 2003), which translates to one in every three cancers diagnosed being a skin cancer [1]. In the UK alone, 12 800 people were diagnosed with malignant melanoma (the most serious type of skin cancer) in 2010, rising to 14 509 new cases reported in 2013 [3]. The cost of skin cancer treatment in England in 2008 was estimated to be in the range of £106–112 million and is predicted to increase to approximately £180 million by 2020 [4]. The increase in skin cancer incidence is thought to be, in part, related to a cultural tendency for more sun exposure, as well as the increased use of sunbeds and, importantly, the inadequate use of sunscreen lotions [3,5].

**Figure 1.**
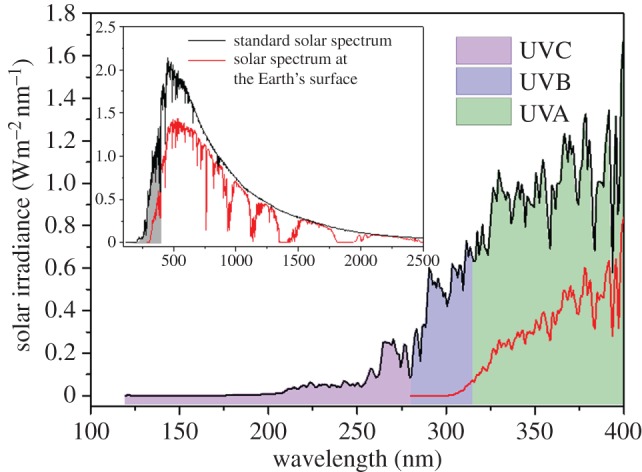
Wavelength regions of UVA (green), UVB (blue) and UVC (violet) radiation in the standard solar spectrum (black line) and the solar spectrum at the Earth's surface, after atmosphere effects (red line). The inset shows the total solar spectrum, with the UV region highlighted (grey shading). Solar spectra raw data obtained from the National Renewable Energy Laboratory (NREL) website [12]. (Online version in colour.)

Like all other types of cancer, skin cancer is a complex problem, the causes of which are not yet fully understood. Nevertheless, ultraviolet (UV) radiation has been consistently shown to be a carcinogen, involved in both direct and indirect DNA damage [6–10], despite its essential role in maintaining plant and animal life on the Earth [11]. UV radiation is classified according to wavelength as UVA (400–315 nm), UVB (315–280 nm) or UVC (280–100 nm), as represented in figure 1 [13]. While the amount of UVC that reaches the Earth is negligible [12], as it is absorbed by the ozone layer in the stratosphere [14], the amount of UVA and UVB radiation at the Earth's surface (approx. 220 µW cm^−2^ on a cloudless day, globally) is chemically significant [15,16].

Both UVA and UVB are capable of causing erythema (sunburn), which in itself is thought to be triggered by DNA damage. It is estimated, however, that approximately 1000 times more UVA radiation is necessary to cause the same level of damage as compared with UVB [17]. Despite being more prominent at ground level (as it is not absorbed as efficiently as UVB by the atmosphere) [18,19], UVA radiation is considered to be less carcinogenic than UVB radiation, because it does not interact with DNA as extensively [20–23]. UVB radiation can be directly absorbed by DNA [21], producing highly mutagenic photolesions, such as cyclobutane pyrimidine dimers and pyrimidine 6–4 photoproducts [20,24]. Even though these errors in the DNA sequence can be repaired by excision repair pathways [20], those which fail to be repaired will result in UV signature mutations and, potentially, carcinogenesis [25,26]. UVB is, therefore, considered to be the most significant type of radiation in the induction of skin cancers [27,28].

While there are natural protection mechanisms against UV-induced DNA damage (vide infra), the prevalence of skin cancer cases makes it clear that artificial enhancement of these mechanisms through man-made sunscreens is necessary. The active agents present in current sunscreens can be categorized into two main groups: physical blockers and chemical absorbers [29]. Physical blockers are substances which reflect or scatter UVA/UVB, such as titanium dioxide and zinc oxide nanoparticles (which, however, also absorb UV radiation) [30]. These species are usually regarded as inert and non-toxic [31], but they are also used in other industries as photocatalysts (for example, in water treatment) [29]. Photocatalytic activity could potentially present a problem in sunscreen formulations and, therefore, there are certain methods designed to reduce it. However, the effectiveness of these methods has yet to be established and their implications on sunscreen safety have not been explored [29]. While this is a considerable problem that requires addressing, this review will focus on the chemical absorbers: molecules which absorb UVA/UVB radiation and thus supplement the skin's own sunscreen molecules, such as melanin pigments.

**Figure 2.**
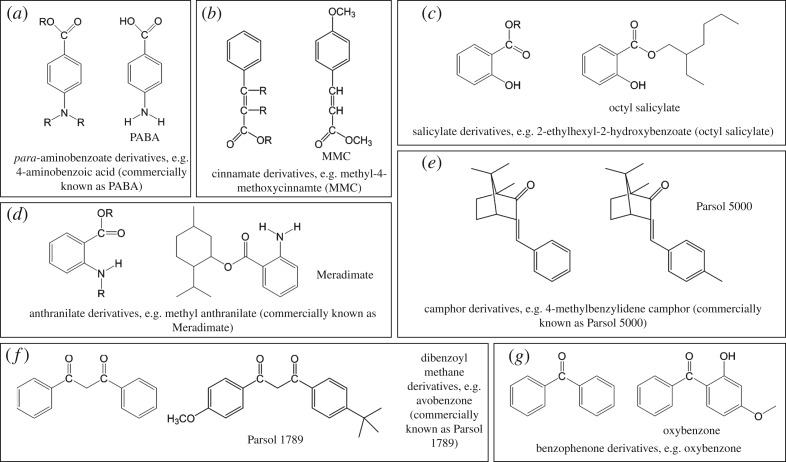
(*a*–*g*) The seven main categories of most sunscreen active absorbing ingredients and representative examples of each.

The chemical absorbers commonly used in the sunscreen industry fit roughly into the seven categories shown in figure 2 (alongside representative sunscreen filter molecules): these are (i) *para*-aminobenzoate derivatives, (ii) cinnamate derivatives, (iii) salicylate derivatives, (iv) anthranilate derivatives, (v) camphor derivatives, (vi) dibenzoyl methane derivatives, and (vii) benzophenone derivatives [29,32,33]. The conjugated systems present in all these molecules allow for strong UV absorption [34–36]. However, no single molecule will provide photoprotection across the entire UVA and UVB range, so chemical absorbers are usually used in conjunction with each other [37] in order to ensure that the sunscreen complies with established regulations, such as sun protecting factor (SPF) values and UVA/UVB absorption ratios [38,39].

Sunscreen formulations are tested for their photoprotective capability by measuring how long they delay the appearance of erythema in treated skin when compared with an untreated sample [40,41]. In addition, and as with any other cosmetic product, sunscreen lotions are tested for dermatological compatibility, in terms of their allergenic and photoallergenic potential [42,43]. It has been reported that organic sunscreen filters can act as photosensitizers, i.e. induce an allergic reaction after absorption of UV radiation; however, the exact mechanisms of photoallergy are still unknown [43].

**Figure 3.**
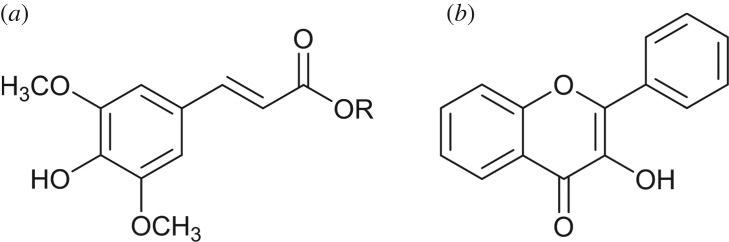
Two main types of plant sunscreen: (*a*) sinapate derivatives and flavonoids, of which (*b*) flavonol is an example.

Particularly in the context of sunscreen development, it is also informative to analyse the mechanisms responsible for photoprotection in natural sunscreens. In much the same way that human skin requires melanin pigments to provide protection against UV damage [44], other living organisms also have an intrinsic need for photoprotection and hence have developed their own photoprotective mechanisms [45,46]. In plant species, UVB radiation was found to have a number of damaging effects, such as growth inhibition, disruption to photosynthesis and transpiration, and general damage to DNA, proteins and membranes [47,48]. In addition, studies have found that plants which lack the ability to efficiently produce certain phenolic molecules, such as sinapate esters and flavonoids (figure 3), are more vulnerable to UVB-induced damage [49]. These molecules are, therefore, believed to provide photoprotection to plant species [50,51]. For example, in *Arabidopsis thaliana* (*Brassicaceae*), sinapate esters were found to play a major role in the attenuation of the adverse effects caused by excess UVB radiation [52]. The intrinsic photoprotective properties of these molecules may be instructive towards the design of the next generation of artificial sunscreens; hence, understanding their photodynamics is of particular relevance. As such, a discussion of these chemical absorbers will also feature herein.

The phenomena, occurring at a molecular level in synthetic and natural sunscreen species upon absorption of UV radiation, which are overlooked by the current sunscreen testing methods, could not only lead to photoproducts but also result in long-lived excited states, both of which have the potential to trigger photosensitivity [53]. Unravelling the photodynamics occurring in sunscreen molecules upon UV absorption will provide insight into their photochemistry and photophysics. This information may then help to identify and assess potentially harmful relaxation pathways and aid a guided development of a new generation of more efficient sunscreens.

**Figure 4.**
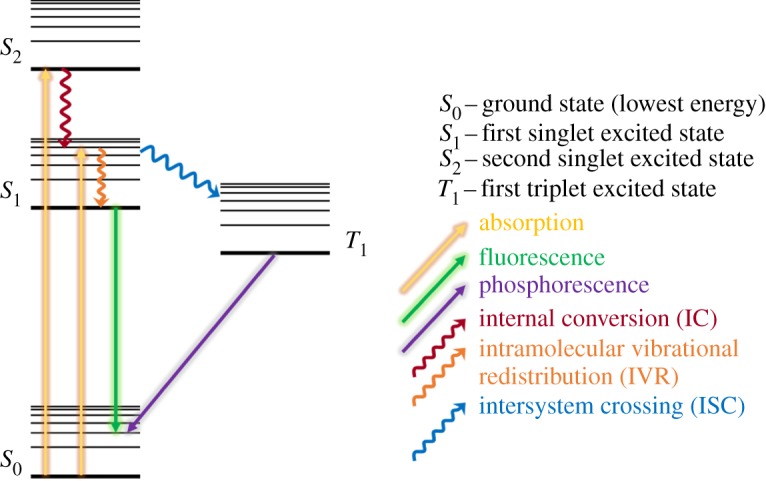
Simplified Jablonski diagram showing some of the possible photophysical processes undergone by a sunscreen molecule after UV absorption. (Online version in colour.)

Upon absorption of UV radiation, a sunscreen will be photoexcited to a higher energy and potentially reactive electronic state [54]. Over time, the system will relax back to the ground (lowest energy) electronic and vibrational state via a number of photochemical/photophysical processes [55,56], such as intramolecular vibrational redistribution (IVR) [57], internal conversion (IC) [58], intersystem crossing (ISC) [58], fluorescence [59] or phosphorescence [60], as summarized in figure 4, where only the photophysical processes are shown for simplicity. Some of these processes may induce damage to the skin if, for example, they result in the production of free radicals or other harmful photoproducts [29,61]. An ideal chemical absorber for a sunscreen lotion should not only strongly absorb UVA/UVB, but should also be capable of dissipating the excess energy gained via mechanisms that cause no chemical change to occur, reforming the molecule in its original state without forming any potentially harmful species.

The more efficient the mechanism by which the molecule returns to its ground electronic state, the less the chance of undesirable photochemistry taking place [62]. The fastest of these relaxation mechanisms typically occur within femtoseconds (fs, 10^−15^ s) or picoseconds (ps, 10^−12^ s) [63]: the timescales of nuclear motion and the breaking (or creating) of chemical bonds. However, other photophysical processes (fluorescence, for example) can take place on the nanosecond (ns, 10^−9^ s) timescale, while phosphorescence can take up to seconds [64]. In these cases, the excited states will persist long enough that there is a higher chance for detrimental photochemistry to occur. An in-depth study on these excited-state decay mechanisms has the potential to unveil any harmful photochemistry that might not be detectable at a macroscopic level if, for example, any long-lived excess energy facilitates damage to DNA.

In the remainder of this review, we will explore the spectroscopic techniques that have recently been utilized in gas-phase studies to initiate this stepwise approach towards unravelling the photochemistry and photophysics of sunscreen filter molecules, as well as providing a synopsis of the contemporary information gathered on this subject to date. These studies provide the initial ground work for an exciting field of research where much is yet to be unravelled, which we briefly expand on in the Summary and outlook.

## 2. Methodologies: frequency- and time-resolved spectroscopy in the gas phase

The advances in laser technology over the past few decades [65] have allowed for the development of a plethora of spectroscopic techniques [66] which enable one to experimentally obtain information on electronic structure and excited-state dynamics which could not be gathered previously. However, owing to the molecular complexity of sunscreen molecules, performing frequency- and time-resolved spectroscopic measurements on them often yields rather intricate spectra and dynamics which may not be unambiguously analysed. Therefore, it is advantageous to employ a bottom-up approach [67,68], wherein a complex system, for example, a large sunscreen molecule, is broken down into its component parts. These components are then studied in the interaction-free environment offered by *in vacuo* gas-phase experiments in order of increasing molecular complexity, building up to the large sunscreen molecule of interest. These studies are then extended into a more real-world scenario [69] by the inclusion of solvents through micro-solvation (gas-phase cluster studies) and solvation (solution phase) as well as increasing the number of interacting species. This bottom-up approach has proved very useful in providing insight, for example into the photostability of DNA/RNA nucleobases and their corresponding chromophore (i.e. UV absorbing) subunits [70–72]. This stepwise approach yields a comprehensive understanding of the photochemistry and photophysics of sunscreen molecules following UV radiation absorption.

This section gives a brief overview of the techniques which have been employed as outlined above to study the sunscreen molecules to be discussed in the exemplar case studies presented in §3.

### (a) Frequency-resolved spectroscopy

A molecule's photochemistry and photophysics are highly dependent not only on the arrangement of its constituent atoms, but also on its electronic and vibrational energy structure, termed *vibronic* structure herein [73]. Therefore, an understanding of a molecule's vibronic structure aids the interpretation of that molecule's photoreactivity [73]. Frequency-resolved spectroscopy techniques can provide a picture of the vibronic structure of a given system by photoexciting molecules to specific vibrational levels and then using various methods to probe these excited states.

High-frequency resolution is provided by narrow bandwidth lasers. The spectral bandwidth of a laser pulse, Δ*ν*, is related to its temporal full width at half maximum (Δ*t*) by the following [74]:

2.1 $$\Delta \nu \geq \frac{K}{\Delta t}.$$

In the above equation, *K* is a constant which depends on the profile of the pulse. To achieve the sub-wavenumber resolution necessary to resolve vibronic (and in some cases rovibronic) states, nanosecond pulses are required [75–78].

Frequency-resolved techniques can be improved by the use of molecular beam technology, which is the current standard for gas-phase experiments [65,66,79]. By seeding molecules in a buffer gas, such as a noble gas, they are collisionally cooled to a vibrational temperature of just a few kelvins [66]. Rapid expansion of the molecules into vacuum ‘freezes’ the sample at these temperatures and a skimmer removes the outer ‘hot’ region of the beam produced. The skimmer, a cone-shaped orifice (see figure 7 for details), separates the source region (where the gas is introduced into vacuum) from the interaction region (where the gas, in the form of a molecular beam, interacts with the laser pulses). This generates a directed flow of cold molecules with a narrow velocity distribution, effectively reducing the Doppler effect [80], resulting in spectral lines with a small Doppler width and hence improving resolution [66].

The main difference between the various frequency-resolved spectroscopic techniques lies in the method used for the detection of the excited states. Some of the possible experimental arrangements are briefly reviewed in the following sub-sections.

#### (i) Laser-induced fluorescence spectroscopy

**Figure 5.**
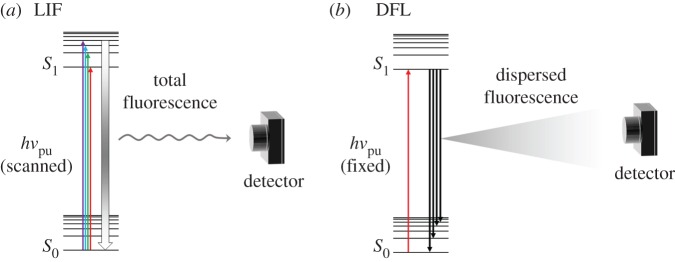
Schematic of LIF (*a*) and DFL (*b*). In LIF, *hν*_pu_ is scanned so that when it is resonant with a certain vibronic state, this state may radiatively decay to any vibrational level in the ground electronic state (*S*_0_, represented by thick gradient arrow). The fluorescence signal is then collected by a photomultiplier. In DFL, on the other hand, *hν*_pu_ is set to a particular vibronic state, from which population may fluoresce to any accessible vibrational level of the *S*_0_ state. The total fluorescence signal is dispersed by a monochromator in order to deconvolute the information on the vibrational levels of the *S*_0_ state, and again collected by a photomultiplier. (Online version in colour.)

In laser-induced fluorescence (LIF) [81,82] spectroscopy, a molecule, initially in its ground vibronic state (*S*_0_), is photoexcited by a pump photon, *hν*_pu_, of variable wavelength, so that specific vibronic excited states are accessed, e.g. in the first excited electronic state (*S*_1_), as represented in figure 5*a*. The radiative decay of these excited states will then result in a fluorescence signal which is collected by a photomultiplier or similar photodetector. From this, a plot of fluorescence intensity versus excitation wavelength, or the excitation spectrum, is produced. Alternatively, *hν*_pu_ may be fixed to a wavelength resonant with a specific vibronic excited state. The resulting fluorescence signal is then dispersed by a grating onto a photodetector so that fluorescence from this state onto the several vibrational levels of the ground electronic state is monitored, as depicted in figure 5*b* [83–85]. This is referred to as dispersed fluorescence (DFL). Fluorescence lifetimes can be measured with either of these set-ups, simply by monitoring the fluorescence signal following photoexcitation over time [86,87]. The temporal resolution of these experiments is then limited by detector response times which, for many common photomultiplier tubes, is approximately 1–10 ns [88].

The high signal-to-noise levels of this technique, afforded by detecting a bright signal on a dark background, coupled with the high intensity of the lasers utilized, afford a sensitivity not achievable in absorption spectroscopy [81]. As fluorescence intensity is proportional to the population of the emitting state, LIF can provide information on the relative population of excited states [82]. However, as LIF is a one-photon process, it is limited by the efficiency of the transitions involved, in both excitation and fluorescence, which are governed by the same symmetry and spin selection rules of any one-photon vibronic transition [34].

As shall become clear in §3, LIF and DFL can be successfully applied to the study of sunscreen molecules in the gas phase, providing valuable insight into their electronic structure.

#### (ii) Resonance-enhanced multiphoton ionization spectroscopy

**Figure 6.**
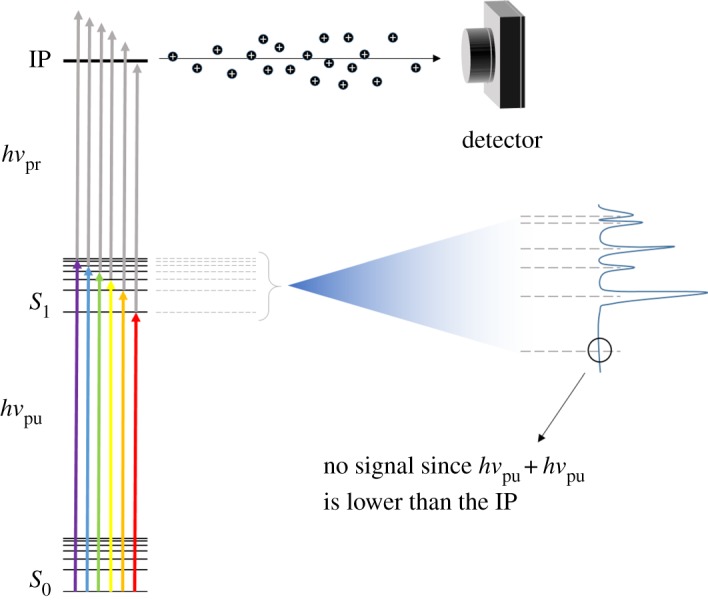
Schematic showing the basic concept behind R2PI. As *hν*_pu_ is scanned, each vibronic state is individually accessed. These states are probed by a second photon, *hν*_pr_, and resulting ions are directed to an appropriate detector (see main text). (Online version in colour.)

Resonance-enhanced multiphoton ionization (REMPI) is a photoionization technique by which multiple photons are used to first resonantly excite (pump) and then ionize (probe) the molecule of interest [76,89]. REMPI schemes are usually referred to as *n* + *m*, where *n* and *m* are integers which correspond to the number of photons, of a single colour, used to pump and probe the system of interest, respectively [89–91]. When different colours are used for the pump and probe steps, this is referred to as *n* + *m*′ [92,93]. While excited states can usually be accessed and probed simply with a (1 + 1) REMPI scheme, also referred to as resonant two-photon ionization, or R2PI, as shown in figure 6, there are cases in which several photons need to be absorbed simultaneously to reach a certain electronic excited state. These multiphoton absorption processes follow an *I^*n*^* power dependence on laser intensity (*I*), where *n* corresponds to the number of photons absorbed [94,95], and they therefore require the high photon densities achievable with lasers.

Regardless of the specific ionization scheme, in a REMPI experiment, the ions produced by the ionization step are monitored as a function of excitation wavelength. This produces spectral lines as each new vibronic excited state is accessed, thus revealing the molecule's vibronic structure. The total ion signal, as a function of probe wavelength, can also be monitored in order to calculate the ionization potential (IP, figure 6) of the molecule under study—the cut-off point, where no ion signal is observed, indicates the point at which the total energy provided to the molecule is no longer sufficient to ionize it [96].

**Figure 7.**
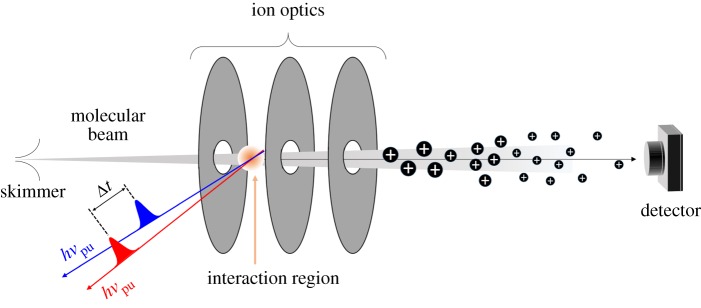
Diagram of a typical gas-phase experimental apparatus, including both a molecular beam and a TOF mass spectrometer. The molecular beam interacts with the laser pulses employed in the experiment. For example, for 1 + 1 REMPI, pump, *hν*_pu_, and probe, *hν*_pr_, laser pulses are necessary, as represented by the red and blue arrows, respectively. The resulting ions are accelerated towards the detector by a set of ion optics. The last section of this set-up consists of a field-free flight tube (see main text), so that ions reach the detector at different times depending on their *m*/*z* ratio. (Online version in colour.)

Ion detection is usually achieved using a time-of-flight (TOF) spectrometer, as shown in figure 7 [97,98]. In simple terms, a TOF spectrometer consists of a vacuum chamber with an ion source at one end, where a molecular beam interacts with the laser pulses, and an ion detector at the other. Ions are accelerated towards the detector by one or more electric fields (continuous or pulsed). Before they reach the detector, the ions go through a field-free flight path, i.e. a region in which no forces are applied, so that the velocity of the ions in this area is dependent on their mass-to-charge (*m*/*z*) ratio. A TOF trace can then be constructed of ion signal against arrival time at the detector. The use of a TOF spectrometer in combination with REMPI spectroscopy thus provides some mass selectivity [89], which allows for contamination from absorbing species other than the molecule of interest that might be present in the molecular beam to be supressed. Like LIF, REMPI spectroscopy benefits from high sensitivity which in this case arises due to the capability to detect all the ions produced by photoionization. In addition, the ionization (probe) step is not as constrained by symmetry selection rules as fluorescence, as photoelectrons will adopt the symmetry required for ionization to take place [99,100].

By careful selection of the temporal pulse width of the lasers used, frequency-resolved (as described above) or time-resolved experiments can be performed in order to gather dynamical information, as shown by the pulse sequences used in figure 7, where the pump and probe are temporally delayed by Δ*t*. This is discussed in greater detail in §2b(i). Some examples of how REMPI spectroscopy, both in a frequency- and a time-resolved context, can be successfully applied to sunscreen molecules will be given in §3.

#### (iii) Double resonance spectroscopy

Double resonance techniques, such as hole burning (HB) or depletion (ion dip) spectroscopies, are useful when the possibility of contributions to the measured spectra from different conformers needs to be taken into account, as R2PI's mass selectivity cannot help distinguish these [89]. In certain cases, contributions from different conformers in vibrational spectra can be distinguished by the analysis of their rotational structure [101–104]. However, especially for larger molecules, the typical resolution achieved with these techniques (vide supra) is not always enough to resolve closely spaced rotational energy levels [34,79,105]. The ability to distinguish between conformers is particularly relevant when dealing with large molecules, such as the ones discussed herein.

**Figure 8.**
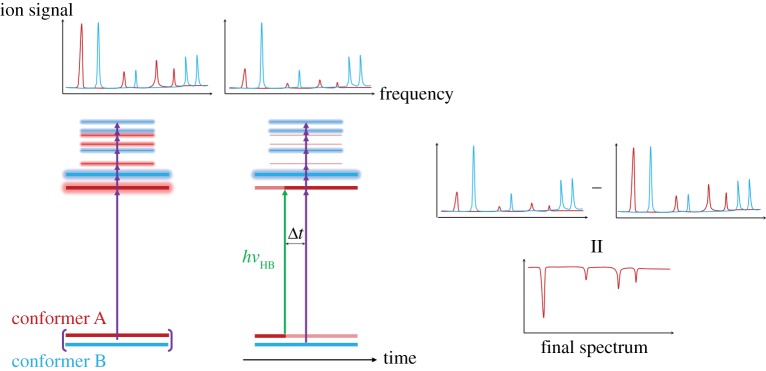
Schematic of HB spectroscopy. The HB laser creates a population hole in the ground state of one of the conformers, so that its contribution to the R2PI spectrum is decreased. Subtracting the total R2PI spectrum from the HB spectrum, therefore, yields the isolated contribution from the selected conformer in the form of negative peaks. Multiple headed purple arrows denote the wavelength scanning of the *hν*_pu_ laser pulse. Various detection techniques (ionization, fluorescence, etc.) can be used. (Online version in colour.)

Double resonance spectroscopy requires the use of three pulses: an excitation, *hν*_pu_, and ionization, *hν*_pr_, laser pulse (as for R2PI) and an extra ‘holeburn’ laser pulse, *hν*_HB_ [89,106]. The term ‘holeburn’ refers to the creation of a population hole in a certain state (e.g. ground electronic state) by exciting the population to a higher vibronic state [106]. At energies close to the excited-state origin, a wavelength that is resonant with a specific transition in conformer A will likely not be so for a second conformer B. Hence, with reference to figure 8, if the wavelength of *hν*_HB_ is set to be resonant with a transition in conformer A, thus depopulating its ground state, the R2PI signal will be reduced when *hν*_pu_ coincides with a resonance in this conformer; however, no analogous signal difference will be observed for conformer B [89]. If the total R2PI spectrum is subtracted from the resulting hole-burning spectrum, the contribution from conformer A can be isolated, with the resulting spectrum consisting of negative peaks.

The same principles apply for depletion spectroscopy, for which the wavelength of *hν*_HB_ is scanned, with *hν*_pu_ fixed on a single transition in one conformer [107–109]. In this case, there will be a reduction in the detected signal whenever *hν*_HB_ is resonant with a vibronic state in this conformer. These techniques, therefore, provide complementary information although one must note that there are differences in the factors that influence ion signal intensities. Depletion spectroscopy monitors changes in ion intensity (as *hν*_HB_ is scanned) compared with a constant pump–probe signal. As such, the signal intensity depends on the absorption cross section of the transition accessed by *hν*_HB_. In HB spectroscopy, on the other hand, as it is the excited state leading to ionization that is being varied, the signal intensity depends on both absorption cross section and also on excited-state dynamics. It is also worth briefly noting that, for both HB and depletion spectroscopy, it is assumed that after photoexcitation by the holeburn laser pulse, population will not return to the lower energy state within the timescale of pump–probe irradiation [110–114].

The HB spectroscopy technique described in figure 8 uses UV radiation in both the hole-burning and pump steps, and is therefore commonly referred to as UV–UV HB spectroscopy. However, as will be demonstrated by the case studies in §3, infrared (IR) radiation can also be used in double resonance techniques, providing conformer selective infrared spectra [112,115].

### (b) Time-resolved spectroscopy

Important photochemical and photophysical processes happen very shortly after the absorption of UV radiation, typically on timescales of the order of femtoseconds to picoseconds (fs–ps, where 1 fs = 10^−15^ s and 1 ps = 10^−12^ s) [34]. The study of such ultrafast phenomena is usually referred to as femtochemistry, a term largely disseminated by the Nobel laureate Ahmed H. Zewail [116]. These ultrafast dynamics may be used to infer how effectively a sunscreen molecule dissipates excess energy and hence its adequacy for use as a photoprotecting molecule.

In order to be able to follow these dynamical processes, a laser temporal pulse width on the order of femtoseconds is needed and currently the technology to generate laser pulses with less than 50 fs pulse duration is widely available [117–119]. However, a higher time resolution can be achieved only at the expense of reduced frequency resolution and such laser pulses are generally incapable of resolving individual vibronic states. Nevertheless, the obvious strength of time-resolved techniques lies in the ability to follow the molecular dynamics occurring immediately after excitation in real-time, achieved by varying the time delay, Δ*t*, between the pump and probe laser pulses, as will be explored further in the next section.

#### (i) Time-resolved ion yield spectroscopy

Time-resolved ion yield (TR-IY) is a technique used to study the dynamics following photoexcitation to an excited electronic state, by monitoring the ion signal resulting from ionization of the excited molecule over time. To achieve this, TR-IY follows the *pump–probe* scheme detailed, for example, by Zewail and co-workers (e.g. [116]), which, in fact, resembles an R2PI scheme (vide supra). Initially, the molecule of interest, seeded into a molecular beam, is photoexcited to an excited electronic state by a pump laser and the system is then allowed to evolve freely over time. After the initial excitation, a probe laser interacts with the photoexcited molecules over a number of time delays, Δ*t*. As the molecule relaxes from its excited electronic state (by any feasible photochemical or photophysical pathway), the population of this state and hence the corresponding parent cation signal will change. Ion detection in TR-IY is carried out as previously described for REMPI experiments, using a TOF mass spectrometer, enabling one to gate on the parent cation (figure 7). By plotting the parent cation signal as a function of time delay, a TR-IY transient is obtained from which the lifetime of the excited electronic state can be extracted. Importantly, by integrating over different mass channels, TR-IY transients of photofragments can also be obtained and the dynamics of their formation extracted. Utilizing such mass selectivity enables one to begin to build up a comprehensive picture of the excited-state decay pathways in operation in a given molecule [68].

The dynamical information obtained from TR-IY thus nicely complements the information gathered from frequency-resolved techniques (and vice versa). It is worth recalling, however, that while frequency-resolved techniques have sufficient resolution to individually access single vibronic states, it is inevitable that the much broader pump pulses used in time-resolved techniques will access several vibrational states within an electronic state. The dynamics and extracted lifetimes correspond, therefore, to a convolution of superposed vibronic states.

Finally, it should be briefly noted that both types of experiment (frequency- and time-resolved) will benefit from insights provided by computational methods [120]. While the specific details of computational studies will not be discussed in the present review, their results will be referred to, where appropriate, in order to inform the discussion of the case studies explored hereafter.

## 3. Case studies

The interest in the excited-state dynamics of UV absorber molecules in a sunscreen context has only recently gained momentum, but there is already a significant collection of important works reported on this subject. This section will comprise an overview of a sample of the recent work carried out on various sunscreen molecules using the gas-phase methodologies described in §2. The authors acknowledge, however, that there are other works which are also relevant to this field of research but were not discussed in this review for the sake of brevity [121]. Similarly, there is the literature describing work carried out on sunscreen molecules and their precursors using the techniques mentioned in this review, but for which the results were not interpreted in a sunscreen context [122]. These studies were, therefore, not included in this review, but they do provide powerful insight towards an understanding of the photophysics of the artificial and natural sunscreen species we will discuss in §3a and b, respectively.

### (a) Cinnamate derivatives

#### (i) Methoxycinnamates

**Figure 9.**
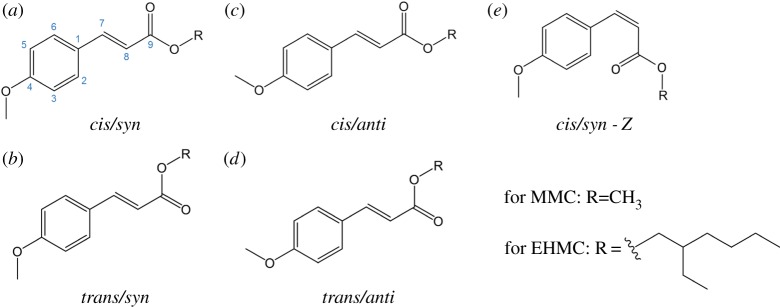
Molecular structures of different rotamers of 2-ethylhexyl-4-methoxycinnamate (EHMC) and methyl-4-methoxycinnamate (MMC). The *cis*/*trans* isomerization occurs around C_8_–C_9_, while the *syn*/*anti* isomerization occurs around C_4_-OCH_3_. *E*/*Z* isomerization occurs around C_7_=C_8_.

*EHMC/MMC*. Many commercial sunscreen lotions use various cinnamate derivatives (figure 2*b*) as UV absorbing species. In fact, one of the most widely used sunscreen agents, 2-ethylhexyl-4-methoxycinnamate (EHMC, figure 9) [123], falls within this category. However, apart from its wide application as a sunscreen, EHMC has been found to undergo potentially harmful chemistry upon UV radiation absorption, such as photoisomerization and release of reactive oxygen species [124–126].

**Figure 10.**
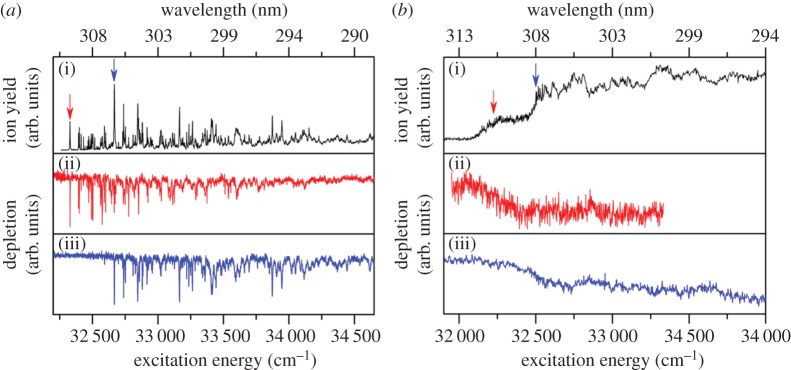
(1 + 1′) R2PI excitation spectra for MMC (*a*, solid black line) and EHMC (*b*, solid black line), where 1 corresponds to the UV excitation pulse and 1′ to a 193 nm ionization pulse. Solid red and blue lines correspond to the separate UV–UV depletion spectra from the *cis* and *trans* rotamers of each molecule, respectively. The arrows indicate the origin bands for each rotamer, to which the molecules were photoexcited when acquiring depletion spectra. Reproduced with permission from reference [127]. (Online version in colour.)

Tan *et al.* [127] studied EHMC in the gas phase using frequency-resolved R2PI and UV–UV depletion spectroscopy. In the same paper, the authors report studies on methyl-4-methoxycinnamate (MMC, figure 9), a simplified version of EHMC, in keeping with a bottom-up approach. The R2PI spectra of both MMC and EHMC are presented in figure 10. Computational simulations were also performed in order to identify the vertical excitation energies and characteristics of the relevant electronic excited states.

Depletion spectroscopy measurements by Tan *et al.* shown in figure 10, suggest that, under the conditions of these experiments, two rotamers of both MMC and EHMC were present: *cis* and *trans* with respect to rotation around the C_8_–C_9_ single bond (figure 9). The *syn* and *anti* pairs are also possible for both MMC and EHMC, with respect to rotation around the C_4_-OCH_3_ single bond, but these were not considered in this work*.* Both the *cis*/*trans* and *syn*/*anti* pairs mentioned are specifically referred to as rotamers, because they interconvert around a single bond and are only distinguishable under conditions of a molecular beam [128–130]. For the benefit of later discussion, it is worthy of note that these molecules can also exist as their corresponding *E*/*Z* stereoisomers (cf. rotamers) with respect to rotation around the C_7_=C_8_ double bond (figure 9).

The origin bands of the lowest ^1^ππ* (*S*_1_) state of MMC were found to be located at 32 328 and 32 667 cm^−1^ for the *cis* and *trans* rotamers, respectively. In EHMC, these bands are located at 32 258 and 32 562 cm^−1^ for the *cis* and *trans* rotamers, respectively. It was also noted that the R2PI spectrum of MMC is more structured than that of EHMC, which was attributed to the increased molecular complexity of EHMC, due to the addition of an ethylhexyl chain.

Tan *et al.* were able to extract the excited-state lifetimes of the ^1^ππ* states for both rotamers of MMC by fitting the R2PI peaks corresponding to the origin bands of each rotamer with a Lorentzian profile. Analysis of these peak-linewidths yielded a reported excited-state lifetime of MMC of 2.9 ps for the *cis* rotamer and 2.0 ps for the *trans* rotamer [127]. The authors noted, however, that these values are lower limits owing to rotational broadening in their experiments. For EHMC, the broad onset of absorption and lack of structure in the R2PI spectrum make the analogous measurements more challenging. Nevertheless, rough estimates of the linewidths of the origin bands in the R2PI of EHMC place its excited-state lifetimes in the sub-picosecond range, which is supported by solution-phase fluorescence lifetime measurements of similar systems reported in the literature [131].

**Figure 11.**
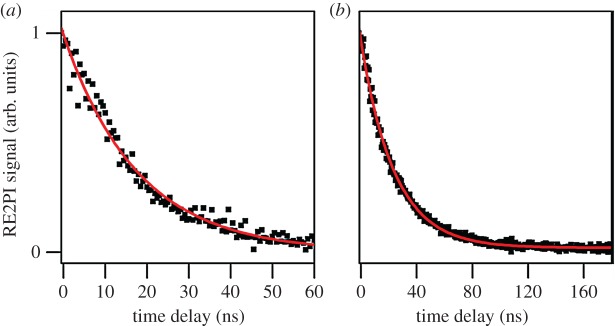
TR–IY of (*a*) EHMC photoexcited at 32 526 cm^−1^ and (*b*) MMC photoexcited at 32 667 cm^−1^. Reproduced with permission from reference [127]. (Online version in colour.)

In an attempt to corroborate these excited-state lifetimes, the authors monitored the time-resolved R2PI signal from MMC and EHMC by varying the time delay between the UV excitation and 193 nm ionization pulses with a time resolution of nanoseconds (figure 11). The recorded TR-IY transients were fit with a mono-exponential function yielding time constants of 24.0 ± 0.2 ns for MMC, and 17.7 ± 0.7 ns for EHMC; much longer than the lifetimes anticipated from linewidth measurements. The observation of two such different time constants in these experiments suggests that two photophysical processes might be occurring in both MMC and EHMC. While the ^1^ππ* states of each of these molecules were found to have a picosecond lifetime, an excited state(s) evidently persists for several nanoseconds. Based on the predictions by other authors [132], Tan *et al.* assigned this nanosecond time constant to a long-lived ^1^nπ* state. This was also reconciled through computational work by the same authors, which places the ^1^nπ* state adiabatically lower in energy than the ^1^ππ* state, in accordance with other cinnamate derivatives [132].

**Figure 12.**
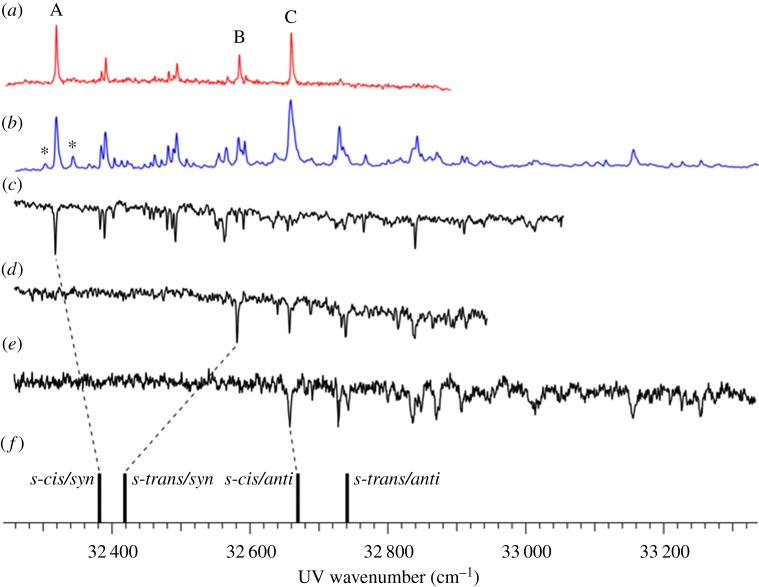
(*a*) LIF, (*b*) R2PI and (*c*–*e*) depletion spectra of MMC under molecular beam conditions. Asterisks (*) in (*b*) indicate hot bands. Calculated electronic transition energies of the different possible conformers (*f*) were also included for comparison with experimental data. Data collected by Miyazaki *et al.* Reproduced with permission from reference [133]. (Online version in colour.)

The findings by Tan *et al.* regarding MMC [127] were expanded on by an independent study by Miyazaki *et al.* [133], who performed similar R2PI and depletion spectroscopy measurements and complemented their interpretation of these results with theoretical studies. Apart from the *cis* and *trans* rotamers previously reported by Tan *et al.* [127], Miyazaki *et al*. [133] were also able to identify the *syn* and *anti* rotamers of MMC (see figure 9 for structures) with the aid of computational studies. As shown in figure 12, Miyazaki *et al.* assigned the experimental bands at 32 328 and 32 667 cm^−1^ to the *cis*/*syn* (figure 9*a*) and *cis*/*anti* (figure 9*c*) rotamers, respectively (cf*.* assignments by Tan *et al.* [127]). In addition, while the peak observed at 32 587 cm^−1^ is assigned to the *trans*/*syn* conformer, the peak that might be expected to correspond to the *trans*/*anti* conformer was not observed experimentally.

**Figure 13.**
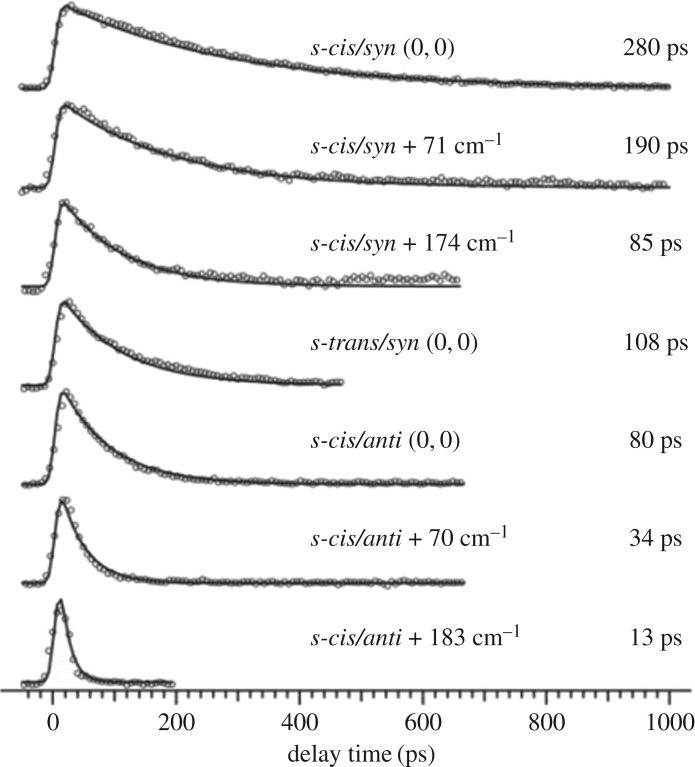
Pump–probe ion signal (circles) and kinetic fits (solid line) of MMC photoexcited to various vibronic levels of the *cis/syn, trans/syn* and *cis/anti* conformers and ionized with 315 nm light. Data collected by Miyazaki *et al.* Reproduced with permission from reference [133].

The time-resolved R2PI signal of MMC following photoexcitation to its *S*_1_ origin was also measured by Miyazaki *et al*. [133] with picosecond resolution (cf. nanosecond resolution in Tan *et al.*'s results [127]), yielding an *S*_1_ lifetime of 280 and 80 ps for rotamers A and C, respectively. The TR-IY transients are presented in figure 13. In addition, the authors also reported these lifetimes to be dependent on excess energy, becoming shorter with increasing excitation energy in *S*_1_ (for each rotamer). While the authors did not reconcile the discrepancies between these results and those from Tan *et al.*'s work, namely in terms of much longer initial time constants and the lack of a long-lived state (vide supra), the latter is very likely owing to a difference in probe wavelength. Miyazaki *et al.* probed for [MMC]^+^ with 315 nm and measured the IP of MMC to be 63 427 cm^−1^ (T Ebata 2016, personal communication). Therefore, the total energy provided to the system (approx. 64 000 cm^−1^) might not be sufficient to ionize from the long-lived, adiabatically lower lying ^1^nπ* state as observed with the 193 nm probe used by Tan *et al.* [127]. Nevertheless, the excess energy dependence of the *S*_1_ lifetimes measured by Miyazaki *et al.* suggests the existence of a potential energy barrier for non-radiative decay via ^1^ππ* → ^1^nπ* IC*.* Importantly, Miyazaki *et al.* have also suggested *E*/*Z* photoisomerization to be a viable non-radiative decay pathway for MMC, occurring in competition with IC from ^1^ππ* → ^1^nπ*.

In summary, both studies (Tan *et al.* [127] and Miyazaki *et al.* [133]) are in general agreement with regard to the photophysical processes occurring in MMC in the gas phase, despite understandable discrepancies in the measured time constants; the time-resolution and probe energies of their respective experiments being probable frontline causes. As a final note, it is also important to discuss a most recent study where Yamazaki *et al.* [134] have presented both experimental and computational data which strongly implicates that triplet states might also be involved in the photophysics of MMC. The authors propose that, upon absorption of UV radiation, and in addition to the aforementioned IC from the bright ^1^ππ* state to the ^1^nπ* state, MMC undergoes ISC from both the ^1^ππ* and ^1^nπ* states into a manifold of triplet states. The long lifetimes of MMC observed by Tan *et al.* [127] could then, according to Yamazaki *et al.* [134], also correspond to the population trapped in these triplet states. These results highlight the role of triplet states in the photophysics of sunscreen molecules, which will be explored later in this review.

*MMC-H_2_O*. The lifetimes of the ^1^nπ* states reported by Tan *et al.* for both MMC and EHMC in the gas phase [127] are orders of magnitude longer than the excited-state lifetimes of eumelanin, one of the skin's natural photoprotective molecules [135]. This would suggest that neither MMC nor EHMC behaves as an ideal sunscreen molecule. However, as sunscreens are administered as lotions, solvent interactions need to be considered. In order to evaluate how the gas-phase spectroscopy and excited-state lifetimes for these molecules might be affected in the presence of solvents, Tan *et al*. [127] preformed micro-solvation studies on MMC, using the same methods described previously.

The R2PI excitation spectrum, obtained by Tan *et al.* resulting from excitation to the origin of the first ^1^ππ* state of the MMC-H_2_O cluster, revealed the presence of four different conformers with origins at 32 106, 32 129, 32 390 and 32 529 cm^−1^, the molecular structures of which were not determined. From the measured linewidths of the bands in the R2PI spectra, there is a factor of two increase from the approximately 2.5 ps ^1^ππ* state lifetime observed for isolated MMC. However, the nanosecond lifetime attributed to the ^1^nπ* state was not observed in the TR-IY measurements of the MMC-H_2_O cluster (cf*.* isolated MMC), with any dynamics now occurring within the instrument response. From these results, Tan *et al.* concluded that IC via ^1^ππ* → ^1^nπ* is no longer an active relaxation pathway for the MMC-H_2_O cluster, and that non-radiative decay to the ground state, mediated by *E*/*Z* photoisomerization is likely the dominant pathway. Recent theoretical work on MMC by Chang *et al.* [136], as well as on related systems [132,137], has also shown *E*/*Z* isomerization to be the dominant relaxation pathway in MMC-H_2_O clusters, while MMC undergoes IC via a ^1^ππ* → ^1^nπ* transition instead. It is well known that polar solvents will significantly destabilize ^1^nπ* states while also possibly stabilizing ^1^ππ* states, resulting in a net increase in the energy difference between these two states [138,139]. Tan *et al.*, therefore, proposed that micro-solvation of MMC is likely to reverse the ordering of its electronic states, effectively inhibiting IC from ^1^ππ* → ^1^nπ*. Even though the same studies were not performed for EHMC, it is likely that these observations would still be valid owing to the relatively unperturbing nature of EHMC's extended hydrocarbon chain. If this is the case, then EHMC would undergo fast relaxation to its ground state by dissipating excess energy into its surroundings, justifying its effectiveness as a sunscreen molecule. Miyazaki *et al*. [133] also studied the MMC-H_2_O cluster to complement the findings of Tan *et al.* [127]. Their R2PI spectrum of MMC-H_2_O revealed that the lowest energy *S*_1_ ← *S*_0_ transition is approximately 200 cm^−1^ lower in energy than that of isolated MMC, being located at 32 106 cm^−1^, labelled *b* and *a*′. Another prominent peak, *b* and *b*′, was observed in the R2PI spectrum of MMC-H_2_O at 32 529 cm^−1^. Comparison between IR–UV depletion spectroscopy measurements (where the hole-burning laser pulse is an IR pulse) and calculated OH vibrational frequencies suggested two conformers of the MMC-H_2_O cluster were present, the first corresponding to a water molecule bridging the carbonyl oxygen and a vinyl hydrogen (*b* and *a*′), the second corresponding to a water molecule bridging the carbonyl oxygen and methyl hydrogen (*b* and *b*′).

**Figure 14.**
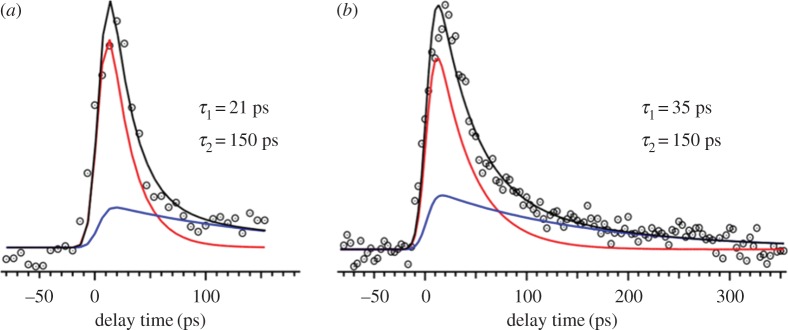
TR–IY (dotted circles) and kinetic fits (black line—total fit, red and blue lines—components) of the MMC-H_2_O complex photoexcited at 32 106 cm^−1^ (*a*) and 32 529 cm^−1^ (*b*), ionized with a 315 nm probe. Data collected by Miyazaki *et al.* Reproduced with permission from reference [133]. (Online version in colour.)

Miyazaki *et al.* [133] performed picosecond TR-IY experiments on the MMC-H_2_O cluster using the same methodology as discussed for the isolated MMC molecule (vide supra). These measurements were taken after excitation to band *a*′ and *b* and *b*′, with both transients modelled with a function consisting of two exponential decays, as shown in figure 14. For band *a*′, the two time constants extracted were 21 and 150 ps, while for band *b*′ the time constants were 35 and 150 ps. Importantly, the shorter time components extracted from the TR-IY transients of both conformers of MMC-H_2_O were shorter than the corresponding time constants for all conformers of the isolated MMC molecule. These observations are in general agreement with the results by Tan *et al.* [127], which showed that micro-solvation of MMC accelerates its relaxation to the ground state. Also in accordance with the suggestions by Tan *et al.* [127], computational work carried out by Miyazaki *et al.* [133] revealed that the micro-solvation of MMC lowers the energy of the ^1^ππ* state and increases that of the ^1^nπ* state. This inversion of electronic states causes the observed red shift in the *S*_1_ ← *S*_0_ transition of the MMC-H_2_O cluster when compared with isolated MMC, as well as the increase of the potential energy barrier for ^1^ππ* → ^1^nπ* IC, which now becomes an unfavourable decay pathway. Potential energy curves also revealed that the energy of the MMC-H_2_O system decreases with increasing dihedral angle towards *E*/*Z* isomerization. *E*/*Z* isomerization is, therefore, the likely relaxation mechanism for MMC-H_2_O, as Tan *et al.* [127] had previously suggested.

As a final note, we briefly refer to another conclusion drawn from Miyazaki *et al.*'s work [133]. While the discussion so far has related specifically to *para-*MMC, in which the methoxy group is positioned in the *para* position with respect to the long side chain, Miyazaki *et al.* [133] also studied *ortho-* and *meta-* MMC (which are not used in commercial sunscreens) in order to evaluate the impact of the substituent position in the photodynamics of MMC. These studies revealed that a radiative decay pathway is now preferred for both *ortho-* and *meta-* MMC [133]. This demonstrates how substituent position may affect the ultrafast dynamics of UV absorbers and, indeed, how an understanding of the photophysics of these systems may help tailor an efficient sunscreen molecule.

#### (ii) Ethyl ferulate

**Figure 15.**
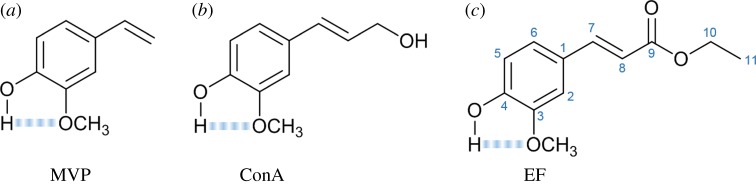
3-methoxy-1-vinylphenol (MVP, (*a*)), 4-hydroxy-3-methoxycinnamyl alcohol (coniferyl alcohol, ConA, (*b*)) and ethyl 4-hydroxy-3-methoxycinnamate (ethyl ferulate, EF, (*c*)). The blue dashed lines represent the H-bonds between the adjacent hydroxy and methoxy substituent groups. (Online version in colour.)

Continuing with cinnamate derivatives, ethyl 4-hydroxy-3-methoxycinnamate (ethyl ferulate, EF, figure 15*c*), a naturally occurring plant product, is another example of a strong UV absorber molecule which is used in commercial sunscreens. Rodrigues *et al*. [140] studied this sunscreen filter molecule using frequency- and time-resolved techniques as well as computational methods. In keeping with a bottom-up approach, two precursor molecules, 3-methoxy-1-vinylphenol (MVP, figure 15*a*) and 4-hydroxy-3-methoxycinnamyl alcohol (coniferyl alcohol, ConA, figure 15*b*), were also studied.

Frequency-resolved R2PI and UV–UV HB spectroscopy measurements carried out for MVP and ConA by Rodrigo *et al*. [141] found that both MVP and ConA are present in two different rotamers, labelled A and B. The two rotamers of MVP have *S*_1_ origins at 32 802 cm^−1^ (A) and 33 525 cm^−1^ (B), while for ConA these origins are located at 32 640 cm^−1^ (A) and 33 445 cm^−1^ (B). Comparison of experimental data with computational results suggests that rotamers A and B for both MVP and ConA correspond to their respective *syn* and *anti* pairs, with respect to the relative positions of the OH substituent group on C_4_ and the vinyl group on C_1_.

**Figure 16.**
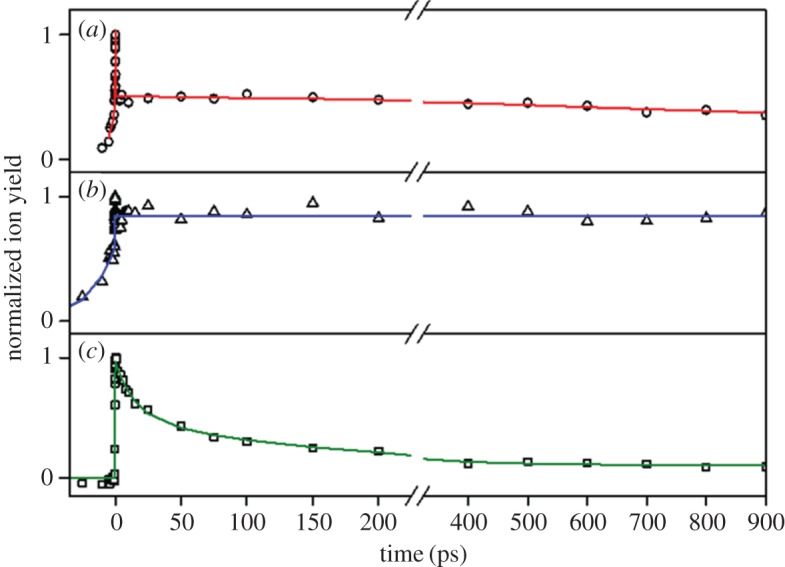
TR–IY (hollow points) and associated kinetic fits of (*a*) MVP, (*b*) ConA and (*c*) EF. Each molecule was excited at the *S*_1_ origin and probed with 200 nm light. Data collected by Rodrigues *et al*. Reproduced with permission from reference [140]. (Online version in colour.)

The TR-IY studies by Rodrigues *et al*. [140] showed that photoexcitation to the *S*_1_(*v* = 0) origin band of the *syn* rotamers of both MVP and ConA directly accesses a long-lived state. This is evidenced by the plateau in the TR-IY transients for MVP^+^ and ConA^+^, figure 16*a,b* respectively, which extend beyond the experimental time window of their measurements (more than 900 ps). The gas-phase studies alone, therefore, suggest that neither of these precursor molecules behave as ideal sunscreens. In EF, however, the addition of the carbonyl moiety was shown to significantly change the behaviour of this molecule when compared with its precursors (figure 16*c* and below). To investigate this further, R2PI and UV–UV HB spectroscopy were performed on EF by Rodrigues *et al.* [140], revealing the existence of two conformers with *S*_1_(*v* = 0) origin bands located at 31 491 and 31 507 cm^−1^. The bandwidth of the laser used for the TR-IY measurements encompasses both conformers (cf*.* single conformer excitation in MVP and ConA [140,141]), thus conformer-specific dynamics could not be obtained and assignment of the bands was not attempted.

Using the R2PI and UV–UV HB spectroscopy data, Rodrigues *et al.* [140] then carried out TR-IY experiments by photoexciting EF with 317.5 nm (approx. 31 500 cm^−1^) radiation and tracking the excited-state dynamics with a 200 nm probe, the transient of which is shown in figure 16*c*. The EF^+^ transient was modelled with a function consisting three exponential decays, returning time constants of *τ*_1_ = 15 ± 4 ps, *τ*_2_ = 148 ± 47 ps and *τ*_3_ > 900 ps. As an accurate lifetime for the long-lived electronic state (*τ*_3_) could not be obtained with the TR-IY set-up used in these experiments, fluorescence lifetime measurements were taken, revealing a lifetime of 6.9 ± 0.1 ns. DFL spectra after excitation to the electronic origin of each conformer were consistent with emission from the *S*_1_ origin.

**Figure 17.**
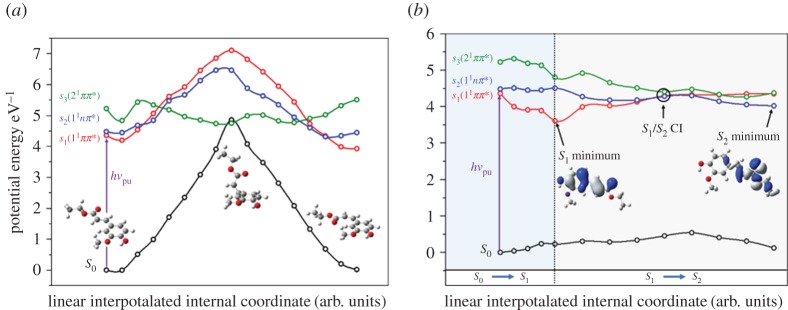
Results of the computational work by Rodrigues *et al*. (*a*) Potential energy cuts (PECs) of the *S*_0_ (black line), *S*_1_(1^1^ππ*) (red line), *S*_2_(1^1^nπ*) (blue line) and *S*_3_(2^1^ππ*) (green line) states of EF along the *E*/*Z* isomerization coordinate are shown. Optimized ground state geometries for each isomer and at the *S*_0_/*S*_3_ conical intersection (CI) are also presented. (*b*) PECs of the *S*_0_ (black line), *S*_1_(1^1^ππ*) (red line), *S*_2_(1^1^nπ*) (blue line) and *S*_3_(2^1^ππ*) (green line) states of EF along linearly interpolated internal coordinates (LIICs). The blue and grey shaded areas represent the *S*_0_ to *S*_1_ and *S*_1_ to *S*_2_ (via *S*_1_/*S*_2_ CI) LIICs, respectively. Optimized geometries and molecular orbitals for the *S*_1_ and *S*_2_ states are also presented. Reproduced with permission from reference [140]. (Online version in colour.)

In the light of these results, computational methods were employed to evaluate possible relaxation pathways for EF. While results from MMC and EHMC (vide supra) would suggest that an *E/Z* isomerization pathway is likely involved in the excited-state dynamics of EF, calculations suggest a very large barrier to such isomerization (approx. 8800 cm^−1^), as shown in figure 17*a*. Similarly, while calculations suggest that an ^1^nπ* state is accessible from the *S*_1_ origin (figure 17*b*), transition into this state would likely result in two lifetimes, as seen in MMC and EHMC (vide supra), rather than the three observed in EF.

Guided by the previous literature, which suggests that the photochemistry of carbonyl compounds is closely linked with the existence of triplet states [54,127,142–149], Rodrigues *et al.* used computational methods to search for such states in EF. These studies revealed three near isoenergetic triplet states approximately 2000 cm^−1^ lower in energy than the *S*_2_(1^1^nπ*) energy minimum (*T*_2_(2^3^ππ*), *T*_3_(3^3^ππ*) and *T*_4_(1^3^nπ*)) and a fourth, lying approximately 10 500 cm^−1^ below these (*T*_1_(1^3^ππ*)). Apart from being close in energy to both the *S*_1_(1^1^ππ*) and *S*_2_(1^1^nπ*) energy minima, these triplet states are also the correct orbital type for favourable ISC, according to El-Sayed's rule; ISC is most efficient for transitions where there is a change in orbital type [150].

**Figure 18.**
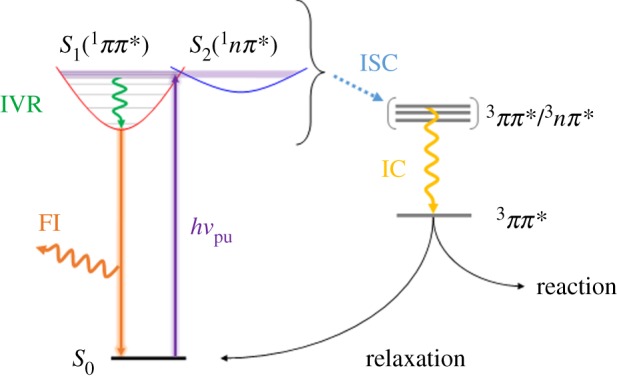
Schematic of the proposed decay mechanisms for excited-state relaxation in EF. Relaxation refers to ISC or phosphorescence back to the ground state; Reaction encompasses such mechanisms as photofragmentation/photocyclization. Note that ‘Reaction’ has been omitted from *S*_1_ for clarity, although it is still a valid pathway. Reproduced with permission from reference [140]. (Online version in colour.)

The complexity of the system under study hindered the unequivocal assignment of time constants to a specific photophysical process. Instead, Rodrigues *et al.* proposed a scenario where multiple processes occur simultaneously, so that the observed time constants correspond to a convolution of relaxation pathways, as illustrated in figure 18. The authors proposed that photoexcitation of EF at 317.5 nm (approx. *S*_1_ origin of both conformers) results in wave packet bifurcation [151,152] to populate both the *S*_1_(1^1^ππ*) and *S*_2_(1^1^nπ*) states, followed by IVR to the respective energy minima of each state and/or ISC into the triplet state manifold. The convolution of these processes, in competition with one another, would then correspond to *τ*_1_ = 15 ± 4 ps. From here, the system evolves via two parallel mechanisms: (i) a convolution of IVR and IC down the ladder of triplet states, happening within *τ*_2_ = 148 ± 47 ps, and (ii) radiative decay of population trapped in the energy well of the *S*_1_(1^1^ππ*) state, corresponding to the measured fluorescence lifetime of 6.9 ± 0.1 ns. Rodrigues *et al.* point out, however, that this is not a unique solution to the assignment of the time constants observed, and that contributions from multiple conformers or photofragmentation pathways cannot be ruled out.

While the authors could not reach a concrete conclusion regarding the relaxation pathways of EF upon UV radiation absorption, this case study serves to demonstrate a number of interesting points. First, these studies suggest that, in the gas phase, EF does not behave as an ideal sunscreen species (nor its precursors MVP and ConA), given the presence of a long-lived excited electronic state. As with the previous case studies involving MMC and EHMC, it is likely that solvent interaction will change this; therefore, solution-phase studies on EF would be highly relevant. Moreover, the case of EF illustrates the complexity of the photophysical processes responsible for the efficiency of sunscreen molecules. When EF is compared with both MVP and ConA, it becomes clear how a simple molecular change can impact the deactivation pathways of a molecule. This also demonstrates how important it is to be able to identify the molecular structures primarily responsible for the dissipation of excess energy, so that future sunscreen species can be tailored for maximum efficiency.

### (b) Sinapate derivatives

**Figure 19.**
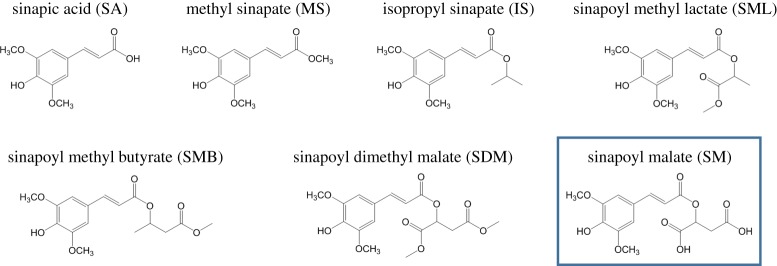
The sinapate series studied by Dean *et al*. [142]. In the lower right corner, marked with a box, is SM, on which this section will mainly focus owing to its role as a natural sunscreen found in the leaf epidermis of certain plant species. (Online version in colour.)

The term ‘sinapate derivatives’ refers to a group of molecules structurally related to sinapic acid, as shown in figure 19. Sinapate derivatives are ubiquitous in nature, being found in fruits, vegetables, grains and oilseed crops, for example, where they exhibit diverse bioactivity [153]. More importantly for the purpose of this review, these species have an important role in plant photoprotection, as discussed in §1. For example, sinapoyl malate (SM), one of the most complex sinapate derivatives, studied by Dean *et al*. [142] is found in the epidermis of *Arabidopsis* plant leaves [154] and it has been shown to be involved in plant photoprotection mechanisms following exposure to UVB radiation [155]. In keeping with a bottom-up approach, Dean *et al*. [142] studied a series of sinapate derivatives of increasing molecular complexity, as shown in figure 19. These molecules were studied with a combination of spectroscopies, including R2PI (one and two colour), UV–UV HB and DFL, as well as computational methods [142]. The following paragraphs present a summary of the results obtained, focusing mainly on SM owing to its prevalence in plant photoprotection.

**Figure 20.**
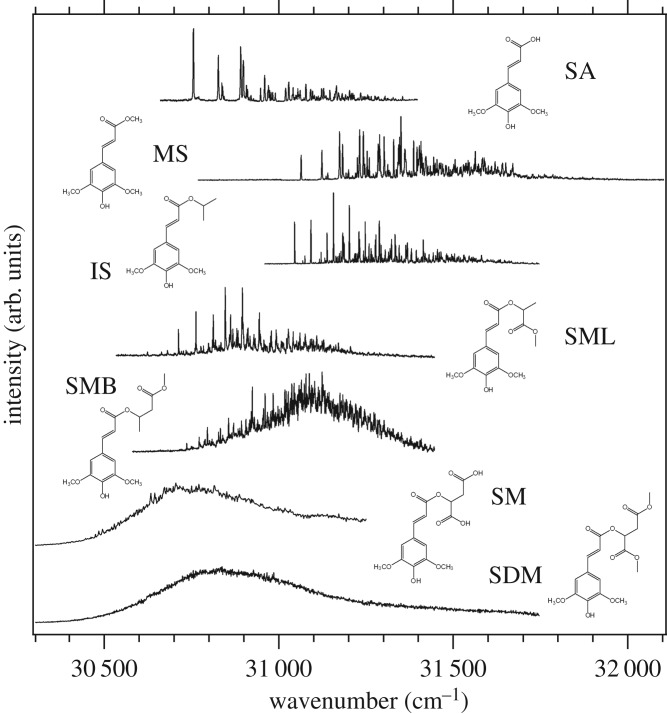
R2PI spectra for the sinapate derivatives studied by Dean *et al*. [142]. While simpler molecules show clearly resolved spectra, increasing molecular complexity introduces vibronic congestion. In the case of SM and SDM, the extensive spectral broadening and congestion results in complete loss of vibronic resolution. Reproduced with permission from reference [142].

All of the sinapate species studied show strong UVB absorption, i.e. all have large oscillator strengths for the *S*_1_(^1^ππ*) ← *S*_0_ transition. The striking differences between the malate containing species, SM and SDM, and the rest of the sinapate series are immediately evident when comparing their corresponding R2PI spectra, as shown in figure 20. While increased molecular complexity results in increasingly congested spectra, as would be expected, the extensive spectral broadening in both SM and SDM appears to be a unique characteristic in this series. The very broad spectra observed for these two species imply effective absorption of radiation across much of the UVB region, which, at first glance, justifies their sunscreen capabilities. Moreover, such spectral broadness may be linked with short-lived excited-state lifetimes, absent in the simpler derivatives studied. Assuming that SM's dynamics involve a fast relaxation to its ground electronic state, this may also justify SM's efficacy as a sunscreen. Therefore, the origins of the unique spectral broadening in SM are of the utmost interest if its sunscreening capabilities at a molecular level are to be understood. This extensive broadening, however, results in featureless spectra that hinder any reliable vibronic analysis; therefore, extracting information from SM's spectra is not straightforward.

While many non-dynamical effects can be responsible for spectral broadening, such as increased molecular complexity or multiple stable conformers leading to congested vibronic spectra, upon comparison with the other sinapate derivatives studied, Dean *et al.* disregarded such reasoning for the featureless spectra in SM and SDM. The less extensive broadening in the structurally similar SML and SMB led Dean *et al.* to rule out IVR as a potential source of the broadening in SM and SDM. Comparison with theoretical calculations also caused the authors to dismiss Franck–Condon activity and/or molecular complexity arguments as viable explanations for these featureless spectra, while IR ion-dip measurements ruled out absorptions from multiple conformers. As such, an alternative explanation for the observed results was sought.

It is well-documented that locally excited states, such as a ^1^ππ* state, can couple with nearby charge–transfer states [156,157]. This can lead to complete spectral broadening by virtue of coupling to a manifold of vibronic levels belonging to the adiabatically lower energy charge–transfer state. In essence, the oscillator strength of the optically bright locally excited state is dispersed across the neighbouring charge–transfer levels. Dean *et al*. [142] carried out theoretical calculations to compute electron density difference maps (EDDMs) of the main singlet excited states of SM. These EDDMs showed that the ^1^nπ* state in SM has partial charge–transfer character, involving a shift of electron density localized around the first carboxylic acid of the malate group and the antibonding orbital on the ring. The existence of a charge–transfer state in SM is supported by fluorescence measurements carried out by Dean *et al*. [142]. These measurements, which were performed in aqueous solution for both SA and SM for comparison, showed a considerable Stokes shift in the emission peak of SM when compared with SA. This suggests that the emitting state is stabilized in a polar solvent, which is a general observation for charge–transfer states, as reported in the literature [158,159]. The fluorescence spectra of SM, therefore, support the existence of a charge–transfer state, as proposed by Dean *et al*. [142].

Despite not having collected any evidence for the existence of long-lived triplet states in SM (owing to experimental constraints), Dean *et al.* also point out the possibility of ISC being involved in its photodynamics. As discussed in previous sections, triplet states have been reported to be active in the excited-state dynamics of carbonyl-containing species [54], and indeed it has been suggested by Rodrigues *et al.* [140] that such states are likely to play an important role in the photodynamics of the natural sunscreen molecule, ethyl ferulate (see §3a(ii)). As triplet states have also been linked to the formation of singlet oxygen [160,161], which would be detrimental to SM's performance as a safe UVB sunscreen, this is another aspect of this molecule's spectroscopy which requires further study.

In conclusion, Dean *et al.* found that, as expected, SM, an important sunscreen molecule in certain plant species, absorbs strongly and broadly across the UVB region. The remarkable broadening of the absorption spectrum of SM was tentatively attributed to a mixing of ^1^ππ* and ^1^nπ* electronic states, the latter having some charge-transfer character. The very low fluorescence quantum yields obtained in solution for SA and SM suggest that non-radiative pathways dominate the excited-state dynamics. These authors encourage the importance of future studies aimed at characterizing the intermediate and final states involved in these dynamics, both in the gas- and solution phase.

## 4. Summary and outlook

While the field of photodynamics of sunscreen molecules is still in its infancy, the work carried out thus far, in both the frequency- and time-domain, has highlighted the wealth of information that may still be gathered, as well as the potential impact of such research to the sunscreen industry. The three case studies reported in this review have revealed a wide range of research opportunities and are likely to encourage further work on the subject. For example, the work by Baker *et al.* [162], which demonstrates how gas-phase studies may be extended into the solution-phase, offers a tantalizing prospect of a plethora of future studies in environments that more closely mimic the surroundings in which these sunscreen molecules are found. Baker *et al.* [162] photoexcited and probed the excited-state dynamics of SA, MS and SM (figure 19) using transient electronic absorption spectroscopy (TEAS) [162–164], in both a non-polar and polar solvent (cf. the gas-phase studies by Dean *et al.* [142], §3b). While frequency-resolved gas-phase studies [142] revealed marked differences in the spectra of these molecules, with SM yielding an exceptionally broadened R2PI spectrum when compared with simpler analogues, the TEAS studies showed remarkably similar data for SA, MS and SM. Importantly, it had been concluded from gas-phase studies that an effective non-radiative process was likely to be responsible for SM's sunscreen properties, but a photoprotection mechanism could not be drawn from the data available. The TEAS studies by Baker *et al.* [162] (guided by the theoretical work of Karsili *et al.* [137] for similar systems) were able to expand on this prediction and provide a detailed picture of the excited-state dynamics occurring in SA, MS and SM. For all three molecules, Baker *et al.* suggest that, after initial photoexcitation to the 1^1^ππ* electronic state, IC to a second 2^1^ππ* state occurs via a 1^1^ππ*/2^1^ππ* conical intersection (CI). This, in turn, is followed by the formation of either the *E* or *Z* conformer of the molecule in the ground state via a second 2^1^ππ*/*S*_0_ CI. Importantly, for all three molecules and in both non-polar and polar solvents, the molecules return to their ground electronic states in tens of picoseconds, making all three good candidates for photoprotection. However, only SM is used in nature as a photoprotective agent, which highlights that there may be other factors that govern the selection of SM as a natural sunscreen agent over the biological precursor SA.

Although the information on the photodynamics of individual sunscreen molecules is valuable (as discussed), commercially available sunscreen lotions are a complex mixture of components (absorbers, scatterers, photostabilizers, fragrances to name but a few). Therefore, the next step within the bottom-up approach would be to evaluate how interactions within such mixtures affect the photodynamics of, say, a sunscreen filter molecule. This would be particularly important for sunscreen filter molecules which populate long-lived states upon absorption of UV radiation when studied in isolation. In a mixture, such long-lived states could be reactive. Indeed, it is well known that some sunscreen blends incorporate the use of photostabilizers [165,166] to counter this. It is, therefore, crucial to explore these types of interactions.

In conclusion, we have seen how frequency- and time-resolved studies in both the gas- and solution phase, as well as computational work, all have the potential to provide crucial insight into the photodynamics of sunscreen molecules. Indeed, as mentioned in the introduction to this review, these techniques (and in particular the bottom-up approach) are also useful in the study of any such photoabsorbing species such as DNA and other biomolecules [70–72]. This information, in turn, can aid in the development of a new generation of sunscreens, tailor-made in order to maximize their efficiency and safety, to make them capable of more effectively tackling the increasing risks of UV radiation exposure in today's society.
